# Age-Related Hearing Loss in Rhesus Monkeys Is Correlated with Cochlear Histopathologies

**DOI:** 10.1371/journal.pone.0055092

**Published:** 2013-02-04

**Authors:** James R. Engle, Steve Tinling, Gregg H. Recanzone

**Affiliations:** 1 Evelyn F. McKnight Brain Institute and ARL Division of Neural Systems, Memory and Aging, University of Arizona, Tucson, Arizona, United States of America; 2 Department of Otolaryngology, University of California Davis, Davis, California, United States of America; 3 Center for Neuroscience, University of California Davis, Davis, California, United States of America; 4 Department of Neurobiology, Physiology and Behavior, University of California Davis, Davis, California, United States of America; UNLV, United States of America

## Abstract

Audiometric hearing deficits are a common symptom of age-related hearing loss (ARHL), as are specific histopathological changes in the cochlea; however, very little data have been collected in non-human primates. To examine this relationship further, we collected auditory brainstem responses (ABRs) from rhesus monkeys spanning in age from 10 to 35 years old, and examined four different morphological features of their cochleae. We found significant correlations between ABR thresholds and the loss of outer hair cells and spiral ganglion cells, but not with the loss of inner hair cells or a reduced thickness of the stria vascularis. The strongest correlation with ABR thresholds was the number of different pathologies present. These findings show that while aged rhesus monkeys experience audiometric hearing deficits similar to that seen in humans, they are not correlated with a single peripheral deficit, but instead with a number of different underlying cochlear histopathologies, indicating that similar histopathologies may exist in geriatric humans as well.

## Introduction

Age-related hearing loss (ARHL), or presbycusis, is a complex auditory disorder that impairs spectral, temporal and spatial auditory perceptions in the aged. One of the more common deficits is a shift in the audiometric thresholds, which is most likely to be manifested peripherally due to pathological changes in the cochlea. It has been hypothesized that different patterns of ARHL emerge in the human as the result of distinct cochlear pathologies [1]–[4], although this dataset is necessarily limited. Subsequent studies in humans have indicated that while there are examples where a single cochlear pathology can account for changes in audiometric thresholds, there are approximately 25% of cases that result from mixed cochlear pathologies [4], and in most cases there are complex interactions between different elements of the cochlea (e.g. see [5]). This is similar to studies in rodents, where either single or multiple pathologies can exist depending on the strain (reviewed by [6]).

How these rodent models can relate to human ARHL remains unclear, and how to relate the results from the different species relies heavily on the questions being addressed and the strain of animals being used. One advantage of broadening the animal models to non-human primates is that the ascending auditory nervous system, particularly the cerebral cortex, is more similar between humans and monkeys compared to humans and rodents, allowing for a better understanding of the cortical contributions to central processing deficits [7], [8]. Accumulating evidence from behavioral and electrophysiological studies suggests that rhesus monkeys also experience ARHL [9]–[13]. Bennett et al. [9] reported age-related changes in both their middle-aged and old groups of monkeys that were primarily due to high-frequency hearing loss. The auditory brainstem response has been used to assess auditory thresholds to clicks [10] and high frequency tones [11], [14] in macaque monkeys. In their most recent study, Fowler et al., [11] found that high-frequency hearing loss increases with age, and that hearing impairments at mid frequencies increased after 21 years of age. These combined results strongly suggest that rhesus monkeys experience at least one form of ARHL. To our knowledge, there has been only one publication on the age-related histopathological changes in the cochlea of the rhesus monkey, and the results from that study were inconclusive. Hawkins et al. [15] showed a progressive loss of hair cells that extended from both the lower base and upper apex with increasing age. They also reported a decrease of spiral ganglion cells that appeared to be primarily restricted to the base of the cochlea, but this pattern was not quantified.

Recently, we have found age-related broadening of spatial tuning curves and impaired gap processing in auditory cortex in two aged rhesus monkeys [12], [13]. This suggests that the age-related histopathological changes in the cochlea of the rhesus monkey may be more extensive that what was previously reported by Hawkins et al [15]. To test this hypothesis and to gain a better understanding of the potential origin of acoustic processing deficits, we directly related auditory brainstem response (ABR) thresholds to clicks and pure tones to the histopathological changes in the cochlea in order to investigate how aging influences audiometric changes in rhesus monkeys. We found that normal aging is strongly associated with decreasing ABR sensitivity and extensive changes across multiple cochlear histopathologies in rhesus monkeys.

## Materials and Methods

### Ethics Statement

All experimental procedures conformed to the National Institutes of Health guidelines for animal use, and were approved by the UC Davis Institutional Animal Care and Use Committee (Protocol number 15783).

### Animals

We used a combination of gross electrophysiological techniques (auditory brainstem response; ABR) and light microscopy to probe the peripheral auditory system for age-related changes in cochlear function and histopathology in 9 rhesus monkeys (*Macaca mulatta*). The age of the monkeys ranged from 10–35 years old. The 10 and 15 year old monkeys were male, while the remaining monkeys were female. These animals were sampled at random from a larger study that is examining the incidence and degree of hearing loss [16]. Six of the nine monkeys were housed at the California Regional Primate Research Center at the University of California of Davis, and were euthanized for unrelated medical reasons. The other three had participated in unrelated experiments and their cochleae were harvested upon completion of a terminal study. All animals were maintained on *ad libitum* food and free access to water. The inclusion criteria were: 1) No known history of receiving ototoxic drugs; 2) No known history of loud noise exposure or traumatic injury to the ear; 3) Auditory brainstem response recorded within 6 months of euthanasia; and 4) Otoscopic examination found no outer ear occlusions or signs of otitis media.

### Auditory Brainstem Response Procedure and Analysis

Each animal was anesthetized with ketamine (10 mg/kg, IM) and medetomidine (0.3 ml/10 kg, IM) and placed in the prone position with their head slightly elevated. The animal’s ear canals were then inspected and cleaned of debris if necessary and to determine the condition of the tympanic membrane. Neither large debris nor otitis media was detected in any of the animals tested. Each animal was given additional injections of ketamine to maintain chemical restraint throughout the procedure, which ranged from 1–3 hours. Following sedation, the skin was wiped clean with alcohol wipes and 0.22 gauge skin electrodes were placed subcutaneously behind each ear, on the forehead, and on the back of the neck [10], [11], [17], [18]. Electrode impedance was checked after placement into the skin, and was below 1 k ohm for all recordings. ABR recordings were obtained by using an Intelligent Hearing System (Smart EP Win USB, Version 3.97) controlled by a laptop computer. Evoked responses were amplified at 100 K times, and our physiological filters were set to pass at 100–1500 Hz. Soft insert earphones were placed in both the left and right ear and connected to a pair of etymotic ER3A transducers for delivery of low frequency stimuli, and then to high-frequency transducers for the 12 and 16 kHz frequency stimuli. The audible range of the rhesus monkeys extends from 26 Hz –42 kHz [12], [19]. We obtained ABRs to clicks and tone bursts (10 msec duration; 2 msec rise/fall trapezoidal envelope) at frequencies of 0.5 kHz, 1 kHz, 2 kHz, 4 kHz, 8 kHz, 12 kHz and 16 kHz. All stimuli were presented binaurally at a rate of 50 stim/sec. ABR waveforms were averaged over a minimum of 1000 repetitions. Clicks and tone bursts were presented at 80 dB pSPL (peak sound pressure level) and decreased in 10 dB pSPL increments until the ABR response was no longer visually identifiable, and then increased by 5 dB pSPL to obtain an estimated hearing threshold. All recordings were obtained in a quiet electrically shielded room or in a double-walled sound booth, and stored for offline analysis. ABR threshold, peak, and latency measurements of waves II and IV were manually scored by two independent raters who were blind to the identity and age of the monkeys. There was greater than 95% agreement between the two raters.

### Histological Processing

Each animal was anesthetized with ketamine (30 mg/kg, IM), and euthanized with a lethal dose of sodium pentobarbital (60 mg/kg, IV). Each animal was then transcardially perfused with saline followed by two fixatives. The first fixative contained a mixture of 4% paraformaldehyde in 0.1 M phosphate buffer (PB; pH 7.4) or 4% paraformaldehyde and 0.5% glutaraldehyde in 0.1 M PB (pH 7.4), and the second fixative contained a mixture of 4% paraformaldehyde and 10% sucrose. The brain was removed from the cranial vault, and then the temporal bone was chipped free. The middle ear was then exposed to puncture the round and oval windows. Each specimen was then submerged in 2% paraformaldehyde and 2% glutaraldehyde in 0.1 M PB and stored in a refrigerator at 4°C. Each cochlea was gently infused through the oval window with 1% osmium tetroxide for 2–4 minutes to reveal the location of the Organ of Corti and to facilitate precise trimming of the remaining temporal bone until only a thin layer of bone remained [20], [21]. Each cochlea was then decalcified in 0.35% EDTA and microwave accelerated [22]. This procedure took 4–8 weeks to reach an acceptable level of decalcification. Following decalcification and additional trimming, one cochlea was randomly selected from each monkey and prepared for whole mount examination, while the opposing cochlea was dehydrated through an ascending series of acetone and then embedded in epon-araldite. Following polymerization, each cochlea was mounted onto a plastic stub and cut in the mid-modiolar plane in 400 µm blocks with a 75 µm thick diamond blade on a radial arm bone saw. Mid-modiolar sections were then cut semi-thinly at 1 µm. Four mid-modiolar sections were collected every 10 µm over 40 µm, mounted on 1×3 glass slides, stained for toluidine blue and basic fuscin for bright field microscopy, cover slipped, and then examined on a Nikon D8000 microscope which was equipped with an MD3-plot x/y stage encoding system (Accustage Inc., Shoreview, MN) and moticam camera which was attached to the computer.

### Data Analysis

We characterized each audiogram based upon the pattern of the ABR threshold shift as a function of age. This was accomplished by calculating the difference between each monkey and the youngest monkey. We then calculated a seven frequency pure tone average (PTA) and a pure tone average loss (PTAL) as a function of age to quantify age-related changes in the ABR threshold functions, and to classify the type of spectral impairment. Next, we used conservative cutoff values that have been assigned to humans [23] to define mild (>30 dB) or moderate to severe (>55 dB) spectral impairments across the low (0.5 kHz), mid (1–8 kHz), and high (12–16 kHz) frequency ranges. Standard light microscopy techniques were used in conjunction with unbiased stereological counting procedures and computerized planimetry to quantify the histopathological changes in each cochlea. We randomly sampled each cochlea due to slight deviations in the angle and position of the mid-modiolar blocks. This strategy, however, hindered our ability to consistently measure the upper apical turn (A3) on every cochlea. We therefore only analyzed the lower and upper turns of the base (B1 and B2), middle (M1 and M2) and the apex (A1 and A2). We measured the density of spiral ganglion cells (SGCs), counted inner (IHC) and outer hair cells (OHCs), and measured the thickness of the stria vascularis (SV). The packing density of SGCs was calculated in each section by randomly overlaying a grid with points that were spaced at 75 µm. A 75 µm unbiased counting frame [24] was then used at every point within the boundary of Rosenthal’s canal throughout the entire modiolus. This strategy allowed us to randomly sample approximately 90–100% of Rosenthal’s canal. Spiral ganglion cells were counted if the nucleolus was present and the nucleus was within and not touching the rejection region of the counting frame. Packing density was expressed as the number of cells per point. IHC and OHC counts were performed on complete or partial hair cell profiles, which included either the cell body or stereocilia. The thickness of the stria vascularis was averaged over 10 orthogonal measurements across the entire extent of the stria vascularis at each level of the cochlea. All values were normalized to the data collected from the youngest monkey to reveal age-related changes in cochlear pathologies. Lastly for each monkey, we quantified the number of pathological features (i.e., density of SGC, IHCs, OHCs, and SV thickness) that were significantly different from the youngest monkey (t-test, p<0.05). This additional assessment accounted for individual variation across animals.

Statistical analyses were performed on the original ABR and the age-normalized data with SPSS, version 19 (Chicago, IL). We used Pearson product moment correlations to examine the relationships between age, ABR thresholds at each stimulus, ABR threshold loss, classified type of spectral impairment, PTA, the density of SGCs, IHC and OHCs counts, and the thickness of the SV. We used stepwise multiple regression analysis to separate the contribution of the cochlear histopathologies on the original and age-normalized ABR, PTA, and classified type of spectral impairment. Explanatory variables were entered in a hierarchical and stepwise manner when appropriate. We used a criterion of p<0.05 to enter and a p>0.10 to remove predictors from the model. We also used t-tests when necessary. Statistically significant relationships and differences were considered significant at p<0.05.

## Results

### Age-related Increases in ABR Thresholds Reveal Multiple Spectral Impairments in Monkeys

**Figure 1:**
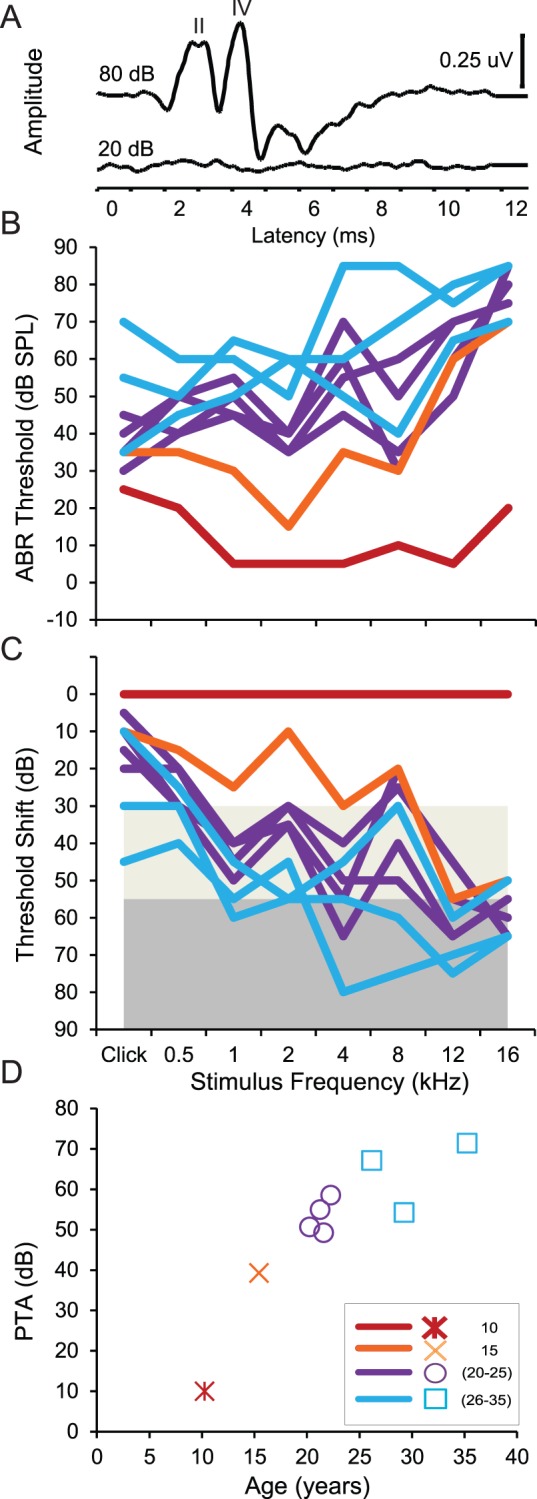
Auditory brainstem response (ABR) measurements of age-related hearing loss. Panel A shows two representative ABR waveforms from the youngest monkey. Waves II and IV are marked. The top trace shows the response to click stimuli presented at 80 dB SPL, which was well above threshold, and the bottom trace shows the response in the same monkey to a 20 dB SPL click stimulus. No obvious response was noted at this stimulus intensity. Panels B-D show the data from each monkey organized by symbols and colorized to represent different age-groups. ABR thresholds (B) and age-related shift in threshold (C) as a function of stimulus and age of the monkey. The seven frequency pure tone average loss (PTAL; Panel D) increases as a function of age, r = 0.88, p<0.01.

We recorded auditory brainstem responses (ABR) to clicks (Figure 1A) and tones in nine rhesus monkeys aged 10 years and 3 months to 35 years and 3 months. Rhesus monkeys age at approximately three times that of humans, so these animals were roughly equivalent to 30 and 105 human years of age. ABR threshold was defined as the lowest stimulus intensity at each specified stimulus to generate an observable wave II and/or IV in the ABR waveform (see Table S1). We found that the ABR thresholds to clicks and pure tones increased with age (see Table 1). Next, we plotted ABR thresholds as a function of the clicks and pure tone frequencies to determine the pattern of loss across the frequencies tested (Figure 1B). Our initial impression of these data was the appearance of increasing thresholds across frequencies, which is a pattern of loss that has been associated with age-related hearing loss in humans and in different strains of rodents [4], [25]. Next, we created ABR hearing loss functions by subtracting the evoked ABR thresholds of the youngest monkey from every other evoked ABR threshold in our study as a function of the stimulus type (Figure 1C). This procedure revealed age-related hearing loss across the frequencies tested, but not as a simple shift of the same audiometric profile across frequencies. What is clear from this analysis is that the higher frequencies were affected to a greater degree compared to lower frequencies, and these deficits also increased with age. Furthermore, we classified the type of spectral impairment as mild if any ABR threshold shift was greater than 30 dB (light gray region of Figure 1C), or moderate-to-severe if the ABR threshold shift was greater than 55 dB (dark gray region of Figure 1C). For example, the 15 year old monkey was classified as having mild high-frequency hearing loss, whereas the 35 year old monkey was classified as the only monkey that had threshold increases that extended from moderate-to-severe for mid and high frequency tones to mild hearing loss for low frequency tones. We found that the remaining monkeys had mild hearing loss for most low and mid-frequency tones, and most had moderate-to-severe hearing loss for the high-frequency tones.

**Table 1:** Correlation matrix between age and ABR thresholds to clicks and tones.

	Age	Click	0.5 kHz	1 kHz	2 kHz	4 kHz	8 kHz	12 kHz	16 kHz
Age	1.00								
Click	0.79*	1.00							
0.5 kHz	0.89**	0.76*	1.00						
1 kHz	0.84**	0.71*	0.91**	1.00					
2 kHz	0.87**	0.57	0.79*	0.90**	1.00				
4 kHz	0.84**	0.75*	0.90**	0.91**	0.75*	1.00			
8 kHz	0.83**	0.96**	0.82**	0.85**	0.70*	0.84**	1.00		
12 kHz	0.74**	0.67*	0.8	0.93**	0.78*	0.87**	0.80*	1.00	
16 kHz	0.69*	0.59	0.86**	0.89**	0.71*	0.83**	0.67*	0.89**	1.00

Note: ** = p<.01, * = p<.05.

One caveat with the above analysis is that it is relatively qualitative. We therefore quantified the type of spectral impairment by calculating the pure tone average (PTA) across the seven tested frequencies and the pure tone average loss (PTAL) on the age-normalized threshold values, and then examined PTAL as function of age (Figure 1D) compared to the qualitative classified type of hearing loss. We found that PTAL was strongly correlated with age (r = 0.88, p<0.01). These combined qualitative and quantitative results strongly suggest that rhesus monkeys experience a pattern of hearing loss that progresses across the audiometric range and increases in severity with age, similar to what is seen in humans.

### Qualitative Observations of the Primate Cochlea

**Figure 2:**
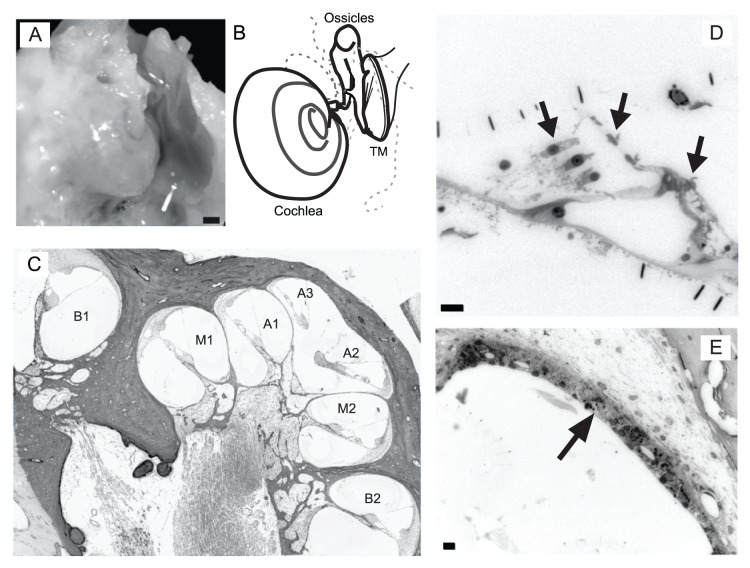
Histology of the rhesus monkey cochlea. This specimen came from the 15 year old monkey. Whole mount (A), illustration (B) and semi-thin mid-modiolar section (C) of the rhesus monkey cochlea. Semi-thin section revealing inner and outer hair cells are shown in D. Arrows indicate stereocilia or soma of inner and outer hair cells. Semi-thin section revealing the stria vascularis are shown in E. Different levels of the base (B1,2), middle (M1,2) and apical (A1,2,3) turns are indicated in panel C. TM = tympanic membrane. Scale bar 1 mm (A), 10 µm (D,E).

Figure 2 illustrates an extracted cochlea from the temporal bone of a rhesus monkey before whole mount dissection (Figure 2A, 2B) and mid-modiolar sectioning (Figure 2C). We gently infused each cochlea with osmium to reveal the location of the Organ of Corti. Whole mount preparations of the opposing cochlea revealed putative myelinated spiral ganglion cell axons that traverse the osseous spiral lamina from the base to apex (Figure S1). This pattern was observed from the base to apex in all nine cochlea specimens. This result suggests that this methodology does not reveal a significant thinning of axons that traverse the osseous spiral lamina, which has been found to be a characteristic of neuritic presbycusis [26]. Additionally, prolonged noise-exposure in monkeys has been shown to result in a frequency specific loss of spiral ganglion cell axons that extends through the osseous spiral lamina [27]. We did not expect to see such degeneration in our sample since one of our inclusion criteria, along with no known administration of ototoxic drugs, was that there was not a significant noise exposure event noted in their medical records. This result suggests that any of the observed pathologies in the cochlea can be attributed to aging and not to significant noise-induced trauma at a single frequency band.

Our initial impression of the mid-modiolar semi-thin sections was that many of the selected features within our analysis deteriorated with age in one or more animals, for example in the Organ of Corti (Fig. 2D) and the stria vascularis (Fig. 2E). One potential caveat with semi-thin sections, however, is that cross sections often appear fractured [28], which may potentially bias hair cell counts. This was accounted for by counting complete or partial hair cells, which included either hair cells or stereocilia (see methods).

### Age-related Decrease of Inner Hair Cells does not Correlate with Changes in ABR Threshold

**Figure 3:**
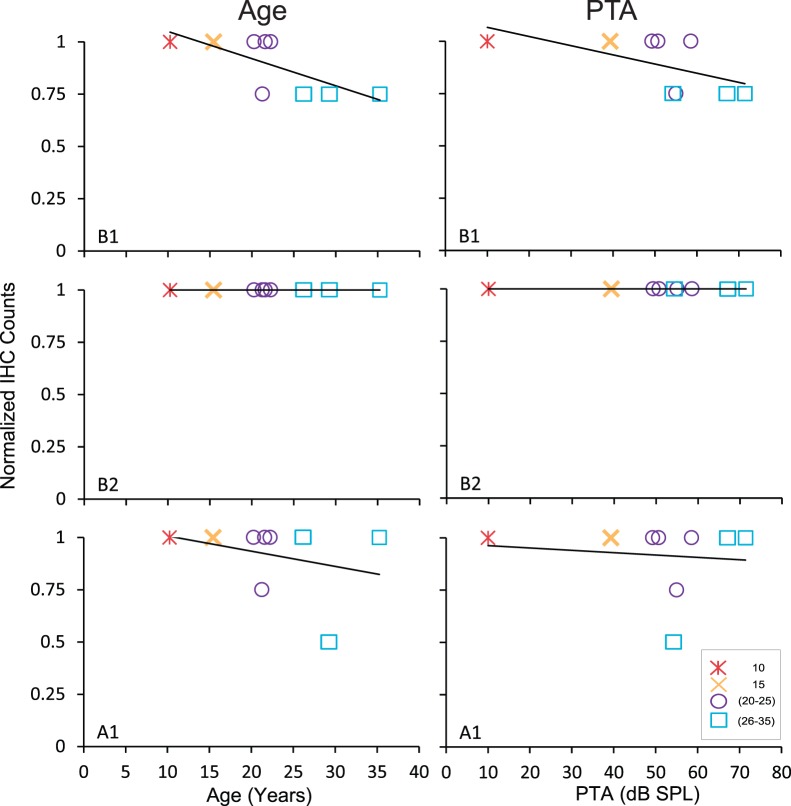
Effect of age and PTA on inner hair cells. Age-normalized inner hair cell counts as a function of age (left) and PTA (right) in the lower base (top), upper base (middle), and lower apex (bottom). A few monkeys had decreased inner hair cells in the lower base and apex, but all were normal in the upper base.

In five out of nine cochleae, we found IHC profiles (Figure 2D) in 95–100% of the sections examined. In the remaining four cochleae, we found reductions of IHCs up to approximately 50% in some of the sections examined. In the next level of the analysis, we analyzed IHCs at different levels of the cochlea to determine if the IHCs deteriorate with age in a selective manner in a particular region of the cochlea, which may ultimately influence the PTA. Figure 3 shows the relationship between the age-normalized counts of IHCs in the lower base, upper base and lower apex as a function of age (left panels) and PTA (right panels; see Table S2 for values for the youngest animal). We found that the number of IHCs in the lower base decreases with age (r = −0.73, p<0.05). The reduction was fairly small with an average IHC reduction of about 12.5% across the sample. We found that the correlations between age and the number of IHCs in the lower base (r = −0.66, p = 0.054) and the upper apex (r = −0.60, p = 0.08) did not meet our criterion for significance. The reductions of IHCs in the lower base and upper apex were also small with an average loss of 9.4% and 6.25%, respectively. Next, we examined the relationship between IHC counts and PTA to determine if the above age-related decreases in counts influenced the degree of spectral deficit. We found that the IHC counts were unrelated with PTA at all levels of the cochlea (lowest r = −0.56, p>0.05). This result suggests that the observed age-related loss of IHCs had, if any, only a negligible effect on the identified audiometric impairments. The contribution of IHCs loss on frequency-specific deficits will be reconsidered in a section below.

### Age-related Decrease of Outer Hair Cells is Associated with Changes in ABR Threshold

**Figure 4:**
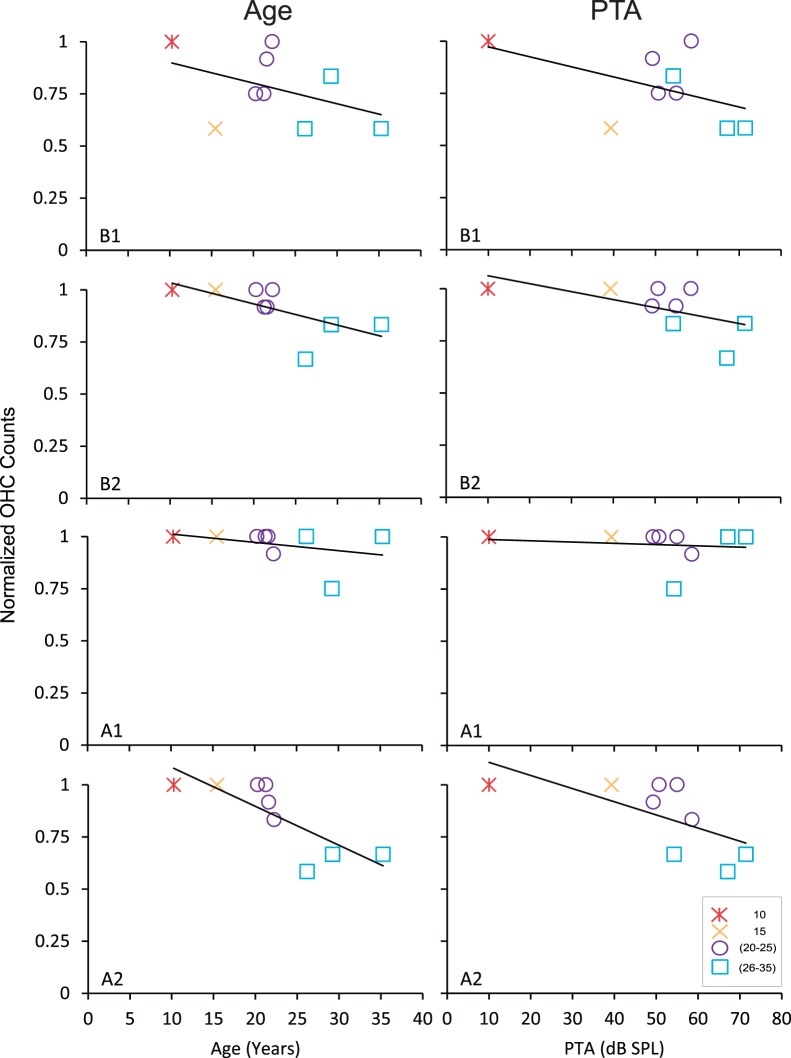
Effects of age and PTA on outer hair cells. Age-normalized outer hair cell counts as a function of age (left) and PTA (right) in the lower base (B1), upper base (B2), lower apex (A1) and upper apex (A2). Many monkeys had decreased outer hair cells, particularly at the lower base and upper apex.

We performed OHC counts to determine the relationship between OHC counts, age and PTA. In six out of nine cochleae, we found complete or partial OHC profiles in 94–100% of the sections examined. In the three remaining cochleae, we found approximate reductions of 45%, 21% and 40% of OHCs in the oldest, second oldest and third oldest monkeys, respectively. We then examined each level of the cochlea independently to determine if the loss of OHCs with age was selective to a particular region of the cochlea. Figure 4 shows the relationship between the age-normalized OHC counts with age (left column) and PTA (right column). Interestingly, the only significant correlation we found between OHC counts and age was in the upper apex, where OHC counts were significantly decreased with age (r = −0.80, p<0.05). The effect was moderately small with an average OHC reduction of about 16.7% across the sample. In the upper base there was a non-significant trend for OHC counts to decrease with age (r = −0.65, p = 0.06). The effect was also small with an average OHC reduction in the upper base of about 10.5% over the sample. Even though we did not find a significant correlation in the lower base, it is noteworthy to mention that the average OHC reduced by approximately 25% across the sample. Next, we examined the relationship between OHC counts and PTA to determine if the above age-related decrease in OHCs in the upper apex influenced the degree of the hearing deficit. Again, we found that decreasing OHC counts in the upper apex was correlated with increasing PTA (r = −0.70, p<0.05). This result suggests that the observed age-related decrease of OHCs is likely to contribute to the identified frequency-specific audiometric impairments in monkeys.

### Age-related and Threshold Related Decreases of Spiral Ganglion Cells

**Figure 5:**
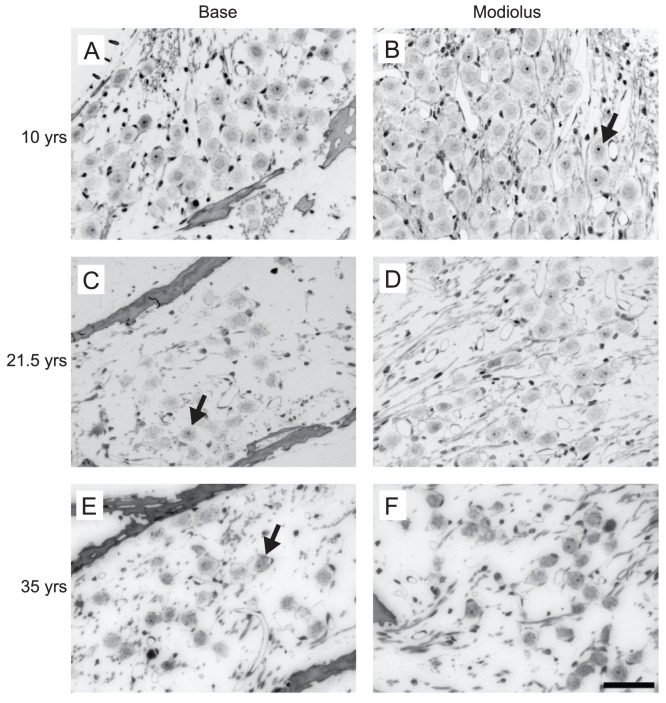
Spiral Ganglion Cell (SGC) loss as a function of age. Photomicrographs of SGCs in the base (left) and modiolus (right) of the 10 (A,B), 21.5 (C, D), and the 35 (E, F) year old monkeys. The packing density of SGCs decreases as a function age and region in the cochlea. Scale bar = 50 µm.

**Figure 6:**
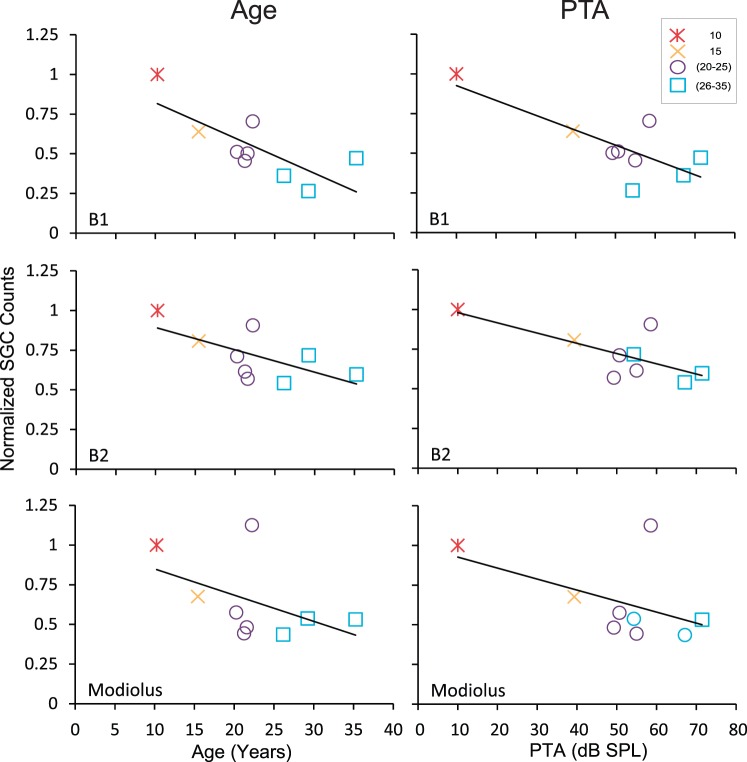
Effects of age and PTA on spiral ganglion cells. Age-normalized spiral ganglion cell counts as a function of age (left) and PTA (right) in the lower base (top), upper base (middle), and modiolus (bottom). There was a clear decrease in SGC as a function of both age and PTA.

The third cochlear morphological feature that we investigated was the packing density of the spiral ganglion cells (SGCs). Figure 5 shows several examples of the observed packing density of SGCs in the lower base and the modiolus of the 10, 21.5 and 35 year old monkeys. The packing density of the SGCs in Rosenthal’s canal appeared to be full in both the lower base and in the modiolus of the 10 year old monkey. However, the packing density was much lower in the base in both the 21.5 and the 35 year old monkey. Quantification of this age-related effect is shown in Figure 6, which along with Figure 5 shows that the packing density clearly decreases with age. In the lower base we found that the density of SGCs significantly decreases with age (r = −0.76, p<0.05), where the packing density was reduced on average by approximately 61%. The packing density of SGCs in the upper base (r = −0.65, p = 0.058) showed a non-significant trend in the same direction as the lower base, with reductions of 32% on average. In the modiolus (r = −0.48, p>0.05), the correlation was not significant although the average reduction in SGC density was 40%. These results suggest that a major age-related effect on the packing density of SGCs is the disproportionate rate of loss of SGCs across the cochlea.

The previous analysis revealed that the packing density of SGCs in the lower base was associated with age, but not in the other regions of the cochlea. It is possible that the packing density of SGCs in the upper base and modiolus are more indicative of changes in threshold across the frequencies tested. Figure 6 shows the relationship between the PTA and age normalized counts of SGCs in the lower base, upper base and modiolus. This analysis showed that that the packing density of SGCs in the lower (r = −0.78, p<0.05) and upper (r = −0.073, p<0.05) base decreases with increasing PTA. This suggests that decreases in the packing density of the SGCs in the lower and upper base, but not at higher levels of the modiolus, are likely to contribute to the identified spectral impairments.

**Figure 7:**
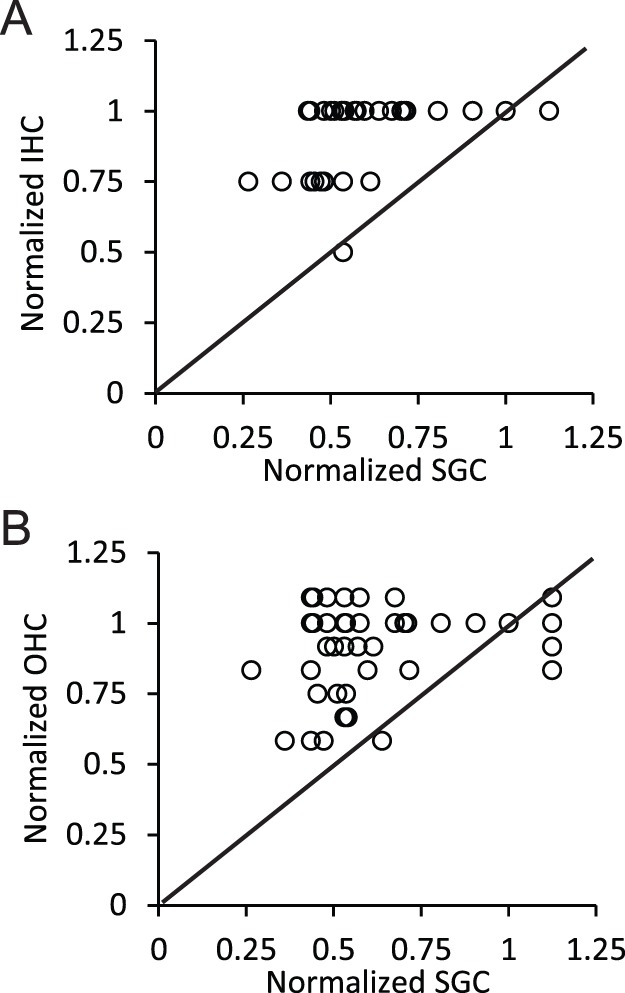
Relationship between the hair cells and spiral ganglion cells. Age-normalized inner (A) and outer (B) hair cell counts as a function of age-normalized packing density of SGCs. The solid diagonal line represents the unity line. Points that fall above the unity indicate a greater loss of spiral ganglion cells compared to the hair cells.

We also investigated whether the age-related decrease in the packing density of SGCs degenerated in parallel with the degeneration of the IHCs and OHCs. Figure 7 shows the relationship between the age-normalized inner and outer hair counts as a function of the age-normalized packing density of the SGCs. The solid diagonal line represents the unity line. A value of 0 indicates the equal loss of either hair cells or SGCs, respectively. Values greater than 1 indicate more cells for that animal compared to the youngest animal, which was usually in the form of either a higher packing density of SGCs or a fourth row of OHCs. Points that land on the unity line indicate that both hair cells and SGCs degenerated at the same rate. Points that fall above the unity line indicate that hair cells degenerated at a slower rate than SGCs and those that fall below indicate the reverse. The majority of points fall above the unity line for both IHCs and OHCs. This indicates that SGCs degenerate at a faster rate than their associated IHCs and OHCs.

### The Thickness of the Stria Vascularis does not Decrease with Age or Spectral Impairment

**Figure 8:**
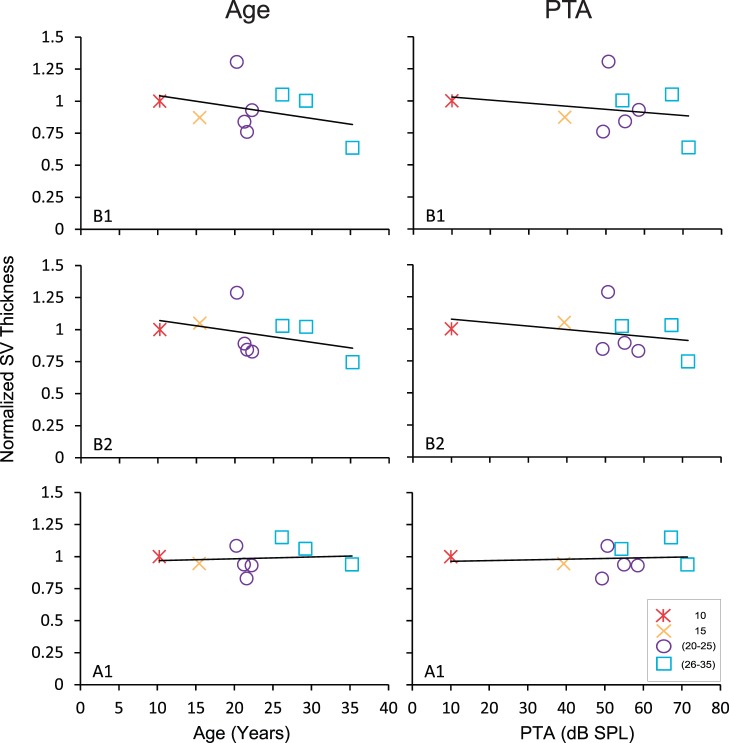
Effects of age and PTA on stria vascularis thickness. Age-normalized stria vascularis thickness as a function of age (left) and PTA (right) in the lower base (top), upper base (middle), and lower apex (bottom). There was no statistically significant correlation across animals.

Finally, we measured the thickness of the SV to determine if a pathological change in the SV is associated with age and PTA. The endocochlear potential has been shown to be inversely associated with the thickness of the SV in the lower base and lower apex [29]. Figure 8 shows the relationship between the thickness of the SV with age and PTA in the lower base, upper base and lower apex. We found that the thickness of the SV remains relatively stable throughout the levels examined in the cochlea of monkeys regardless of age or the degree of spectral impairment (largest r = −0.47, p>0.05). This result does not support the hypothesis that the SV thickness contributes to the identified spectral impairments in monkeys.

### SGC Density and OHCs Contribute to the High Frequency Audiometric Hearing Impairments in Monkeys

The above results indicate that rhesus monkeys experience several age-related cochlear pathologies. We used multiple regression analysis to determine the best predictors of age-related changes in the ABR thresholds. In a step-wise manner we entered explanatory variables that could theoretically predict the ABR threshold of a specific frequency region in the cochlea. For example, we entered the cochlear features in the lower base, the upper base, and the lower middle turn as predictors of age-related change in threshold to the 16 kHz tone stimulus to determine how the highest frequency tested is influenced by these regional cochlear features. For this analysis, we found that the packing density of SGCs in the lower base (b = −0.643, t(8) =  −6.608, p<0.001), and number of OHCs in the lower middle turn (b = 0.596, t(8) = 6.123, p<0.001) contributed to the age-related ABR threshold loss at 16 kHz. In the final model, the packing density of SGCs and the number of OHCs accounted for 95% of the variance in the age-related changes in ABR threshold at 16 kHz (R^2^ = 0.946, F(2,6) = 37.491, p<0.001). Interestingly, adding the most basal turn did not improve this correlation, presumably as this region is normally processing frequencies much higher than 16 kHz in the macaque, where the hearing range extends beyond 30 kHz (see [12], [19]).

The age-related decrease of SGCs contributed to a large proportion of the variance in the age-related decrease of the ABR threshold at the highest frequencies tested. It appears, however, that the age-related decrease of OHCs is the primary contributor to the age-related increase of ABR threshold to clicks, and at middle frequencies of 1, 2 and 8 kHz. IHCs and the thickness of the SV contributed a very small percentage to the variance of age-related changes in hearing thresholds. These combined results suggest that the major contributors to age-related high frequency hearing loss in monkeys are the concomitant age-related decline of SGCs and OHCs.

**Figure 9:**
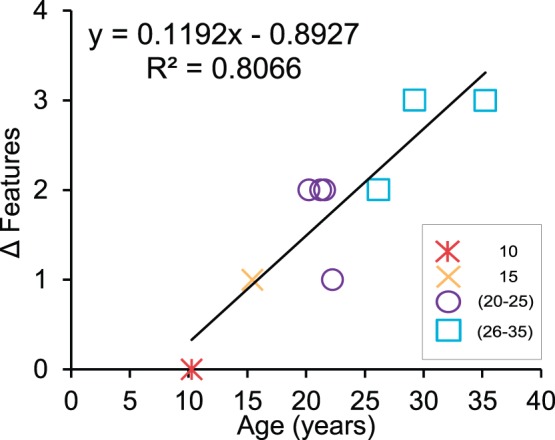
Age-related increase of the number of significant pathological features in the cochlea of the rhesus monkey. There was a statistically significant correlation, indicating that multiple histopathologies occur with natural aging. r = 0.90, p<0.001.

To build upon this analysis, we examined the number of significant age-related differences across the four features in the cochlea that were under investigation. Significant differences were identified by making age-related comparisons after collapsing the data within a single cochlea. For example, the overall packing density was significantly lower in the 15 year old monkey compared to the 10 year old monkey (t-test, p<0.05) while no other differences were found. Therefore, the 15 year old monkey had only one significant age-related difference from the 10 year old monkey. On the other hand, the 35 year monkey had three features that were statistically different relative to the 10 year old monkey. The SV thickness, OHC counts and SGC counts were all significantly lower in the 35 year old monkey (t-test, p<0.05). The number of significant pathological differences was strongly correlated with age (r = 0.90, p<0.001), and PTA (r = 0.78, p<0.05). This result is shown in Figure 9 (see Figure S2 for individual data). This suggests that the number of age-related pathological features increases with both age and the severity of hearing loss. We then used multiple regression analysis to determine if age-related changes at a particular frequency could predict the number of significant pathological changes in the cochlea. For this analysis, we found that the age-related increase of ABR thresholds at 2 kHz can be used as a predictor of the age-related increase in the number of significant pathologies in the cochlea (b = 0.836, t(8) = 4.023, p<0.01). In the final model, threshold changes at 2 kHz accounted for 70% of the variance in the number of significant age-related pathological changes in the cochlea (R^2^ = 0.698, F(1,7) = 16.184, p<0.01). This result suggests that a frequency-specific deficit at 2 kHz is the best predictor of the extent of cochlear histopathological changes in monkeys.

## Discussion

Rhesus monkeys are widely used in studies that explore system level questions in the auditory cortex (see [7], [8], [30], [31]), and in aging [12], [13]. Studies of the auditory system in aged rhesus monkeys are sparse, and the origins of the reported processing deficits are unclear. Using the auditory brainstem response to assess auditory function, this and previous studies have demonstrated increases in audiometric thresholds across the frequency spectrum with age [11], [16]. In the current study this was observed as an initial age-related increase which was restricted to high frequencies and slowly progressed with age across the entire frequency spectrum. Similar patterns have been reported in humans and in animal models of presbycusis [25], [32], [33]. These similarities led us to examine and characterize the relationship between age-related audiometric shifts to age-related cochlear histopathological changes in rhesus monkeys.

We found several correlations between age, increases in ABR thresholds, and distinct decreases in the cochlear histopathology of rhesus monkeys. The first important finding is that the decline in spiral ganglion cells and outer hair cells accounts for most of the decreases in high and mid frequency hearing, respectively. A second important finding is that the number of different cochlear histopathologies is very strongly correlated with age. These results suggest that rhesus monkeys experience spectral hearing impairment with mixed cochlear pathologies and indicate that a similar condition of multiple cochlear pathologies could underlie age-related audiometric deficits, as well as temporal and spatial processing deficits, in humans [34], [35].

### Parallels of Age-related Hearing Loss in Humans and Monkeys

Early studies of ARHL in humans indicated that specific audiometric shifts were related to a distinct cochlear histopathology [1], [2], [4]. Our understanding of ARHL, however, was slightly obscured by the inclusion of temporal bone specimens with potential histopathological confounds that were generated by means other than natural aging (for review see [5]), and by studies that did not find a relationship between audiometric changes and cochlear histopathology [3], [36]. Recent studies of human temporal bones, however, provide a different picture due to more rigorous evaluations of patient data, and stringent inclusion criteria. Nelson and Hinojosa [5] found that the severity of the hearing loss in the aged was associated with the degeneration of IHC, OHCs, SGCs and the stria vascularis. This also applies to monkeys as this same association was found in the present study. Additionally, Nelson and Hinojosa [5] found that the extent of cochlear degeneration of the above four elements was highly associated with audiometric changes at 8 kHz in humans. In monkeys, we found that extent of cochlear degeneration of these same four elements was strongly associated with audiometric changes at 2 kHz. If these findings translate to other species, then audiometric shifts at a specific frequency can be used to identify animals with multiple cochlear pathologies. This will greatly aid in participant or specimen selection for future studies on ARHL.

### Age-related Changes in the Auditory Brainstem Response of Rhesus Monkeys

We found that the ABR thresholds increased as a function of age regardless of stimulus type (Figure 1). By defining mild and moderate-to-severe hearing loss across the low, mid, and high frequencies, we found that the severity of loss in rhesus monkeys increases as a function of age. The identified patterns of hearing loss showed a transition from downward to gentle sloping ABR audiograms as a function of age, which is one of the more common conditions in humans. We did not observe elevated and flat hearing loss as has been described previously in humans [32], as well as in some instances in a larger population of monkeys [16], which is likely due to the relatively small sample of monkeys in the current study. There have been only a few studies in rhesus monkeys that probed for ARHL with ABRs. In an early study on how caloric restriction affects ARHL, ABR responses to clicks were found to only have a trend to increase with age [14], and that it increases at an approximate constant rate of 1.2 dB per year. In a follow-up study on how caloric restriction affects ARHL longitudinally, ABR thresholds increased at different rates that varied with the stimulus type and age [11]. Importantly, Fowler et al. [11] found both linear age-related increases in hearing threshold, and curvilinear age-related increases that began around 21 years of age. The results of this study indicate that the rate of hearing loss may not be constant, and that the underestimated 1.2 dB decrease may be frequency specific. Our results show age-related increases in ABR threshold to the click stimulus at an approximate rate of 1.8 dB per year. Our results also demonstrate that the rate of loss decreases at different rates across the tone frequencies tested, especially in the low and mid tone frequencies.

### Age-related Histopathological Changes in the Cochlea of Rhesus Monkeys

We found several age-related histopathological changes in the cochlea of the rhesus monkey. This included an age-related decline in the packing density of SGCs, IHC loss that was primarily found in the lower base and OHC loss that extended from the lower base. For hair cells, Hawkins et al., [15] reported a similar but more extreme pattern of loss in the lower base. This difference can be accounted for by differences in our strategies to analyze the cochlear specimens. We performed our analysis on mid-modiolar cross sections that randomly sampled the tissue whereas Hawkins et al. [15] cut the Organ of Corti away from the modiolus into several millimeter sections from the apex to base, and then performed mid-modiolar cross sections on the remaining tissue. For SGCs, we found an age-related decline in the packing density that extends from the lower base into the modiolus. Although qualitative, Hawkins et al. [15] reported a similar pattern in the lower base in their sample of three aged monkeys. They also found in one middle aged animal that SGC loss throughout the modiolus was extensive, which suggests that SGC loss may be independent of hair cell loss. We found that the packing density of SGC decreased with age, but at differential rates in the lower base, upper base and the modiolus. This suggests that SGC loss may be regionally specific and then progress over the lifespan of the animal. This loss is also independent from hair cell loss, which is a pattern that has been reported in humans and in some rodents [4], [37]–[39]. For example, Zilberstein et al. [40] highlighted this independence by showing that SGCs survive for weeks following the targeted ablation of IHCs in a mouse strain that lacks a thiamine transporter. The likely culprit behind SGC loss found in monkeys is the accumulation of mild to moderate noise overexposure over the lifespan in a similar fashion that has been shown in rodents [41]. Interestingly, we saw no difference in the spiral ganglion cell axons across age using whole-mounts of osmium stained cochleae (Figure S1). This indicates to us that this method is not sensitive enough to reveal the level of SGC loss seen in these animals, and that more sensitive anatomical techniques are required to detect a corresponding loss of SGC axons [42].

Lastly, we found that the stria vascularis was not associated with a specific audiometric shift in hearing, which is congruent with previous observations of the stria vascularis in aged rhesus monkeys [15]. Stria vascularis thickness has been reported to be associated with the endocochlear potential, and scales with age [29]. In our hands, the thickness of the stria vascularis did not significantly change with increasing age in a similar fashion that has been reported in rodents [25]. We also found that the thickness of the stria vascularis was not significantly correlated with age-related changes in the ABR thresholds as a function of frequency. This may be due to several reasons. The lack of observing changes in strial thickness does not necessarily imply that rhesus monkeys do not experience changes to their audiogram based upon strial pathology. Our sample was fairly small and predominately female, and strial ARHL has been linked to genetic factors [43]. In addition, inspection of Figure S2 shows that there were several cases where SV thickness was reduced, but did not reach our criteria for statistical significance. An increased sample size, or inclusion of even younger monkeys may reveal differences in SV thickness with age. Future studies in rhesus monkeys could identify monkeys with flat elevated hearing loss, and then examine their relatives for similar ARHL outcomes to determine the extent of strial pathologies on flat elevated hearing loss in monkeys.

### Summary and Functional Implications

In summary, the results of this study indicate that, very much like humans, rhesus monkeys suffer a progressive hearing impairment that increase in severity with natural aging. Our analysis suggests that increases in threshold at the low to mid frequencies can be accounted for by decreases of OHCs rather than to changes of the stria vascularis. Additionally, the loss of SGCs throughout the cochlea may impair neural signaling from the cochlea to its central targets, and inevitably auditory cortex. Importantly, the strongest correlation with hearing loss and aging was an increase of the number of cochlear pathologies. If this translates to humans, then this finding suggests that a single remedial strategy may not be effective at treating ARHL as it progresses over the lifespan. This also indicates that, while the ABR thresholds as a function of stimulus frequency could be similar in shape, the cochlear deficits could vary considerably (Figure S3). This indicates that either ABR thresholds, or behavioral audiograms, may show similar deficits but may be due to very different cochlear pathologies, and therefore further information would be necessary when attempting to find the most effective treatment. These findings also implicate a central compensatory mechanism that may be used to enhance the reduced activity from a significantly smaller population of spiral ganglion cells. The ability of these different mechanisms to compensate for this reduced activity may underlie the different perceptual consequences, such as deficits in localizing sounds and understanding speech, between individuals with apparently similar peripheral hearing loss.

## Supporting Information

Figure S1 **Osmium stained whole mounts from a 15 and 35 year old monkey.** Osmium staining reveals apparently normal spiral ganglion axons through the osseous spiral lamina in young and old animals. This pattern was observed in every specimen regardless of age.
(EPS)

Figure S2 **Summary profile of the age-related cochlear histopathological changes.** The data from each of the 8 animals is normalized by the youngest. A value of 1 indicates no difference from the youngest animal, larger values indicate a greater number than the youngest animal and smaller numbers indicate a decrease compared to the youngest animal. SG: spiral ganglion cell density; IHC: inner hair cell counts; OHC: outer hair cell counts; SV: stria vascularis thickness.
(EPS)

Figure S3 **Auditory brainstem response thresholds to clicks and tones in the 21.25 (solid line), 21.58 (dashed line), and the 35.25 (dotted line) year old monkeys.** Two cochlear histopathologies were found in middle aged monkeys. Both had a significant reduction of SGCs. The 21.25 year old also had a reduction of IHCs, while the 21.58 year old had a reduction in the thickness of the stria vascularis. The 35.25 year old had three cochlear histopathologies. These were reduction in the thickness of the stria vascularis, reduction of SGCs and a reduction in the number of OHCs.
(EPS)

Table S1 **Summary demographics and ABR threshold to clicks and tones.**
(DOCX)

Table S2 **Densities and thickness of the observed cochlear elements in the 10 year old monkey.**
(DOCX)
