# Supplementary material for: Age-Related Hearing Loss in Rhesus Monkeys Is Correlated with Cochlear Histopathologies
Source: PLoS One. 2013 Feb 4;8(2):e55092. doi: 10.1371/journal.pone.0055092 (PMC3563598; doi:10.1371/journal.pone.0055092)
Supplement: Table S1 — Summary demographics and ABR threshold to clicks and tones. (DOCX) [file pone.0055092.s004.docx]

Table S1. Summary demographics and ABR threshold to clicks and tones.

|  |  |  | ABR Threshold (dB SPL) | | | | | | | |
| --- | --- | --- | --- | --- | --- | --- | --- | --- | --- | --- |
| Monkey | Age | Sex | Click | 0.5 kHz | 1 kHz | 2 kHz | 4 kHz | 8 kHz | 12 kHz | 16 kHz |
| Y1 | 10.25 | M | 25 | 20 | 5 | 5 | 5 | 10 | 5 | 20 |
| Y2 | 15.42 | M | 35 | 35 | 30 | 15 | 35 | 30 | 60 | 70 |
| M1 | 20.25 | F | 30 | 40 | 45 | 40 | 60 | 30 | 60 | 80 |
| M2 | 21.25 | F | 45 | 40 | 50 | 35 | 55 | 60 | 70 | 75 |
| M3 | 21.58 | F | 35 | 50 | 45 | 35 | 45 | 35 | 50 | 85 |
| M4 | 22.25 | F | 40 | 50 | 55 | 40 | 70 | 50 | 70 | 75 |
| O1 | 26.17 | F | 55 | 50 | 65 | 60 | 60 | 70 | 80 | 85 |
| O2 | 29.25 | F | 35 | 45 | 50 | 60 | 50 | 40 | 65 | 70 |
| O3 | 35.25 | F | 70 | 60 | 60 | 50 | 85 | 85 | 75 | 85 |
