# Supplementary material for: Age-Related Hearing Loss in Rhesus Monkeys Is Correlated with Cochlear Histopathologies
Source: PLoS One. 2013 Feb 4;8(2):e55092. doi: 10.1371/journal.pone.0055092 (PMC3563598; doi:10.1371/journal.pone.0055092)
Supplement: Table S2 — Densities and thickness of the observed cochlear elements in the 10 year old monkey. (DOCX) [file pone.0055092.s005.docx]

Supplemental Table 2. Densities and thickness of the observed cochlear elements in the 10 year old monkey.

| Cochlear region | Whole mount | IHC density (mm)* | OHC density (mm)* | SGC density (mm^2^) | SV thickness (µm) |
| --- | --- | --- | --- | --- | --- |
| Lower Base | No Axonal Gaps | 125.00 | 375.00 | 169.88 | 23.94 |
| Upper Base | No Axonal Gaps | 125.00 | 375.00 | 124.55 | 24.51 |
| Lower Modiolus | No Axonal Gaps | 125.00 | 343.75 | 222.42^ǂ^ | 25.87 |
| Upper Modiolus | No Axonal Gaps | 125.00 | 375.00 |  | 22.64 |
| Lower Apex | No Axonal Gaps | 125.00 | 375.00 |  | 23.51 |
| Upper Apex | No Axonal Gaps | 125.00 | 375.00 |  | 21.77 |

* Estimated from sample. ǂ Density in the modiolus extends from the lower modiolus upwards to the upper apex.
